# Performance of Azure-winged magpies in Aesop’s fable paradigm

**DOI:** 10.1038/s41598-020-80452-5

**Published:** 2021-01-12

**Authors:** Yigui Zhang, Cong Yu, Lixin Chen, Zhongqiu Li

**Affiliations:** Lab of Animal Behavior and Conservation, School of Life Sciences, Nanjing University, Nanjing, 210023 Jiangsu China

**Keywords:** Ecology, Zoology

## Abstract

In this study, the improved Aesop’s fable paradigm—a series of experiments originally used to test whether some animals understand the causality associated with water replacement—was used to explore the cognitive ability of Azure-winged magpies (*Cyanopica cyanus*). Experimental results on causal cue tasks showed that the Azure-winged magpies prefer water-filled tubes over sand-filled tubes, heavy objects over light objects, and solid objects over hollow objects. However, they failed to notice the diameter and water level of the tubes. They also failed to pass the counterintuitive U-shaped tube task in arbitrary cue tasks. Our results demonstrated that Azure-winged magpies have a certain cognitive ability but not an understanding of causality, a characteristic comparable to that of other corvids. Moreover, Azure-winged magpies exhibited the ability of training transfer and analogical problem solving from the perspective of cognitive psychology. We believe that object-bias has little effect on Azure-winged magpies in this study. We can conclude that the Azure-winged magpies partially completed the tasks by trial-and-error learning.

## Introduction

As the most cognitively advanced species, human have a variety of complex cognitive abilities, such as causality, perception, memory, attention, thinking and imagination. We usually refer to the functional relationship between an event (cause) and a second event (result) in which the latter event is considered to be the result of the previous event as causality. It is not difficult to imagine that animals with certain causal cognitive ability can solve many survival problems^1^. Therefore, many researchers have used different methods for different species to explore whether animals have certain causal cognitive abilities^2^.

“The Crow and the Pitcher” is one of the famous fables of Aesop. It tells the story of a thirsty crow who drinks water by progressively throwing small stones into the bottle to raise the water level^3^. This story demonstrates simple causality. Given that stones are denser than water, throwing them into the bottle will raise the water level until the crow can drink it. Numerous researchers designed several experiments based on this fable (water displacement) to explore animal cognition and understanding of causality. Such experiments are called the Aesop’s fable paradigm. This paradigm has been adopted to study causal cognition in many species, including humans. For example, the first category involves four species of birds of the family Corvidae, namely, rooks (*Corvus frugilegus*, this research shows that rook can use tools to solve complex problems^4^), New Caledonian crows (*C. moneduloides*, these studies claim that New Caledonian crows has a certain understanding of displacement^1,5,6^), Eurasian jays (*Garrulus glandarius*, they claim that the Eurasian jay is able to complete tasks because of rewards and causal clues^7^), Western scrub-jays (*Aphelocoma californica*, this study to explore whether their cognitive abilities are restricted to a caching context^8^). The second category involves great-tailed grackles (*Quiscalus mexicanus*) of the family Icteridae^9^. Not only did they partially pass the experiment in the Aesop’s fable paradigm but also adjusted (but not completely change) their previous behavior as conditions changed (such as when the initially more functional object became no longer functional).The third category involves kea (*Nestor notabilis*) of the family psittacidae^10^. They claimed no evidence of causal understanding of water displacement and highlight that such an understanding is not required for solving Aesop’s Fable by testing kea parrots on their understanding of water displacement. The fourth category involves raccoons (*Procyon lotor*) of the family procyonidae^11^, raccoons performed differently than corvids and human children did in previous studies of Aesop's Fable, and researchers found raccoons to be innovative in many aspects of this task. The fifth category involves humans^12^. Several researchers observed children aged 4 to 10 years. They found that the performance of children aged 5 to 7 years was similar to that of New Caledonian crows, and children over 8 years old were able to successfully complete all tasks (including U-shaped tubes) in the first trial. Some of these researchers claimed that subjects exhibit an understanding of causality in Aesop’s fable paradigm^1,4,6,7^.

Two other hypotheses may explain the success of these experiments on establishing the understanding of causality: the object–bias hypothesis and the feedback hypothesis. The object–bias hypothesis states that when subjects are faced with the choice of objects rather than tubes, if they had a preference for certain functional objects before every task began (i.e., the experience of all subjects using stones in the initial training may affect their subsequent choices.), then they have a greater probability to pass the object selection experiment in Aesop’s fable paradigm^13^. Indeed, performance in object-choice Aesop's Fable tasks are influenced by object biases in New Caledonian crows but not in human children^14^. By comparison, the feedback hypothesis asserts that when subjects are faced with the choice an object or a tube, the successful subjects notice the change in the position of the reward after dropping the stone and then repeat the action until they obtain the reward^3,15–17^. However, subjects did not succeed in L-shaped and U-shaped tasks with feedback cues^7^. Nevertheless, these hypotheses cannot establish that subjects show understanding causality in Aesop’s fable paradigm. Although the Aesop’s fable paradigm has several shortcomings in confirming whether animals have the ability of causal understanding, it can be modified accordingly to address these problems^5,13,18^. A recent study reported that Aesop’s fable paradigm cannot distinguish between trial-and-error learning and causal reasoning^19^. Hennefield et al. used meta-analytic techniques to suggest that objects learned from successful (but not failed) behaviors, that is, corvid causal reasoning in the Aesop’s Fable paradigm is driven by trial-and-error learning^20^. Birds exhibit trial-and-error learning in Aesop’s fable paradigm but not causal understanding^18^. Although the current criticisms of Aesop’s fable paradigm focus on its inability to establish that subjects understand causal relationships, some species undeniably show a certain cognitive ability (i.e., tool-use) in Aesop’s fable paradigm experiments. So, when the subjects face Aesop's fable paradigm, do they show the ability to understand or learn causality? Bhat and Mohan’s study will give us the answer. This study emphasized that reasoning and learning must always exist simultaneously, that is, whether it is natural or artificial cognitive agent, it must continuously to accumulate and develop throughout learner’s life^21^.

Azure-winged magpies (*Cyanopica cyanus*) are mainly distributed in eastern Asia, with strong clustering and sociality, highly developed communication ability, and keen alertness^22^. As a member of the family Corvidae, Azure-winged magpies may also have considerable intelligence and cognitive level. Previous studies mainly focused on their cooperative reproduction and mutual assistance behavior^23–26^. Researchers have recently shown interest in the physical and social cognitive ability of Azure-winged magpies. They have explored the cognitive ability of Azure-winged magpies through string-pulling tasks^27^, mirror tasks^28^, and cooperation and prosocial behavior^29^. These studies showed that Azure-winged magpies have certain physical cognition, including but not limited to perception and spatial cognition. Evidence from above studies indicated that Azure-winged magpies demonstrate innovative problem solving and behavioral flexibility, consequently, Azure-winged magpies are a clever species capable of overcoming novel challenges. Aesop’s fable paradigm remains an excellent paradigm for exploring animal physical cognition. It requires subjects to choose functional options based on the displacement of water. We attempted to improve this paradigm by reducing object–bias interference and explore the role of visual feedback. Based on a previous study^18^, we developed a new method for data analysis to explore the cognitive ability of Azure-winged magpies from the perspective of cognitive psychology-a science believes that cognition is information processing, including the entire process of encoding, storing and extracting sensory input, which studies cognitive processes, such as attention, perception, representation, memory, thinking, and speech. In this study, we quoted the data on another species belonging to family Corvidae^1^ and compared their performance to understand the physical cognitive abilities of different bird species and the evolution of cognition. Based on a previous methodology^1^, we conducted six experiments that explore cognitive ability from different angles: water-filled tubes *vs.* sand-filled tubes, heavy objects *vs.* light objects, solid objects *vs.* hollow objects, tubes of different diameters at the same water level, tubes with high water level *vs.* tubes with low water level (same diameters), and U-shaped tubes.

## Methods

### Subjects

Seven adult Azure-winged magpies were named Alina (A), Boyce (B), Carl (C), Denny (D), Joyce (J), Kyle (K), and Sin (S). A and S were females, and the rest were males. A, B, J, and S were captive individuals (3.5 years old), whereas C, D, and K were rescued in the wild (about 2 years old). J participated in mirror and string-pulling tasks^27^, and A, B, and S participated in mirror tasks^28^. K failed the initial training; thus, only six individuals actually participated in the experiments. The Azure-winged magpies were kept individually in the same type of cage (60 cm × 40 cm × 40 cm) and provided daily with drinking water and food. Fruits and mealworms were available every day at 18:00. Multiple branches were provided for the birds to roost, and small ball toys were provided for them to play. During the experiments, the Azure-winged magpies were provided with water only but no food from 10:00 to 14:00 on the same day to increase the motivation. The experiments were conducted from 14:00 to 17:00. They were immediately fed with daily food after the experiments. They had ad libitum access to water.

### Ethics statement

The study was conducted and approved by the *Animal Welfare and Ethical Review Committee of Nanjing University* (IACUC—2003001) and carried out in compliance with the ARRIVE guidelines. All methods were carried out in accordance with the relevant guidelines and regulations. This article does not contain any studies with human participants.

### Initial training

**Figure 1:**
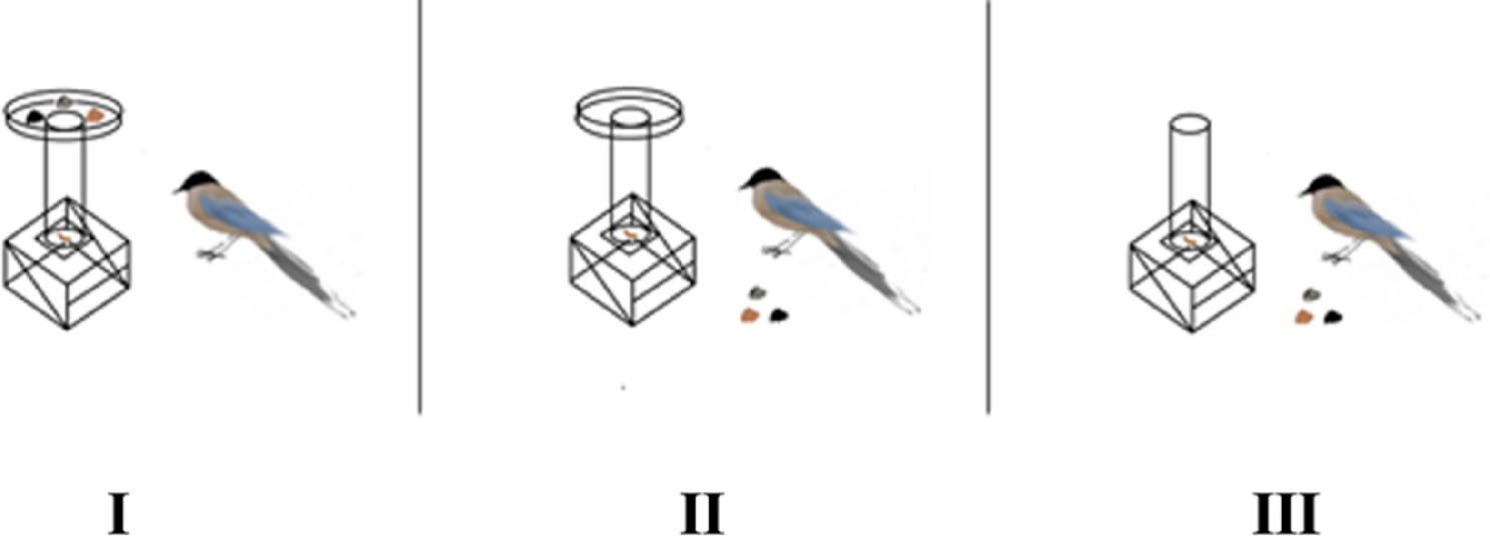
Training device. (**I**) Owing to the stimulation provided by the possibility of obtaining a reward, the Azure-winged magpies used their beak or body to touch the device to make the natural stone in the tray slide into the tube, thereby triggering the mechanism to make the reward fall. If the Azure-winged magpies started to push the stones in the tray into the pipe actively, it will enter stage II. (**II**) After several times, the natural stones were randomly scattered in the cage. If Azure-winged magpies moved the stone onto the tray and repeated stage I or directly put the stone into the tube, it marks the completion of stage I. If not, returning to phase I. (**III**) The tray was removed, and natural stones were randomly selected and scattered in the cage. If Azure-winged magpies directly put the stone into the tube, it marks the completion of stage II. If not, returning to phase II.

**Figure 2:**
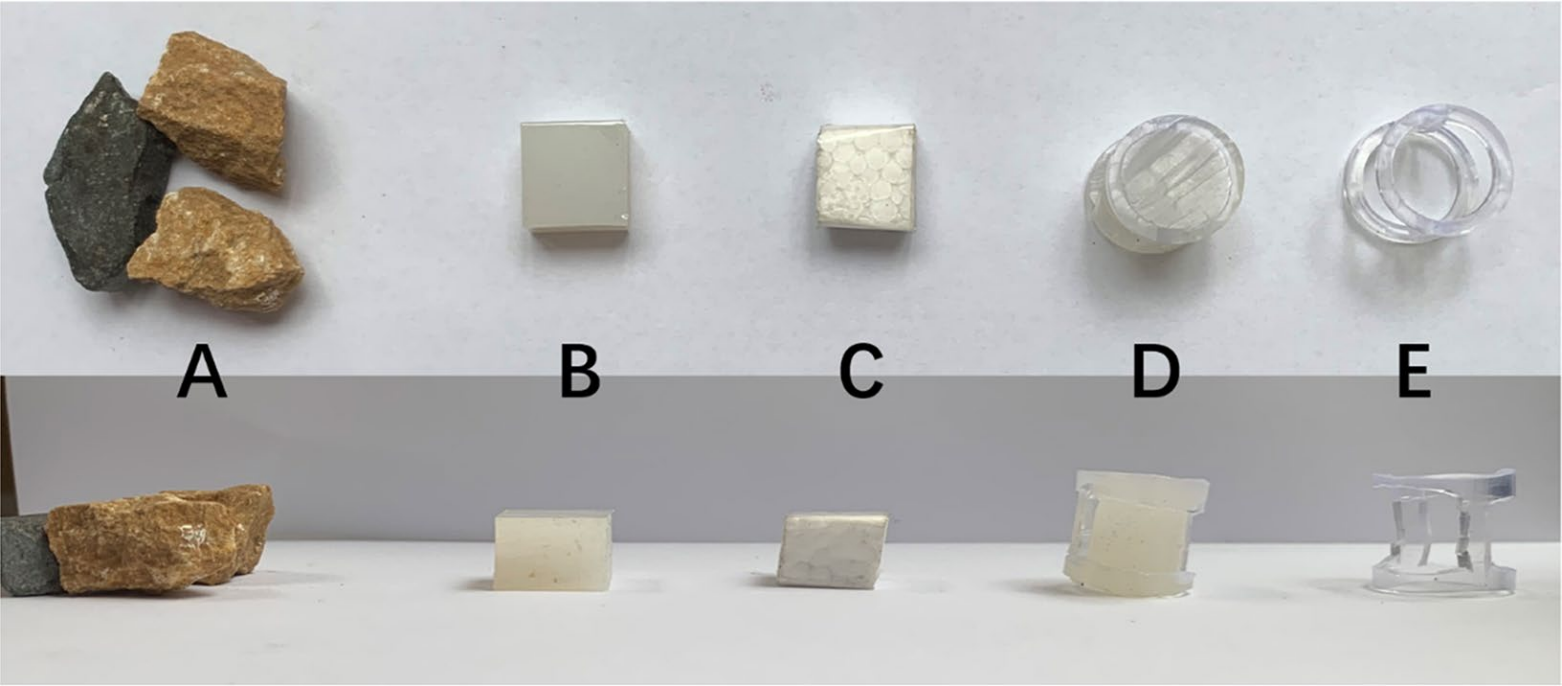
(**A**) Natural stones used in the initial training (8.21 ± 1.05 g). (**B**) Rubber blocks with a higher density than water (3.06 ± 0.03 g). (**C**) Foam blocks with a lower density than water (0.08 ± 0.01 g). (**D**) Solid cylinders (3.77 ± 0.05 g). (**E**) Hollow cylinders (1.04 ± 0.01 g).

The Azure-winged magpies raised in our laboratory and those rescued in the wild would not have encountered a situation similar to putting stones in a tube to raise the water level until they could drink it. Therefore, we used a device (Fig. 1) to train the Azure-winged magpies to exhibit the behavior of putting stones (of unique colors and sizes, Fig. 2) into a tube (only one stone is enough to trigger the device adequately). Individual differences were observed in the transition from I to II to III for each individual (K could not complete the conversion from I to II at all). Nevertheless, they quickly adapted. Given that the transition from phase I to phase II was difficult, phase I was repeated many times. When the Azure-winged magpies successfully repeated stage II 20 times in the same day, the conversion was deemed successful. When the subjects successfully completed phase III 20 times in the same day (Azure-winged magpies directly put the stone into the tube), the subjects were deemed to have successfully passed the training. The training phase was different from that of previous studies^1,5,13,30^ because we did not establish a phase with multiple stones that could trigger the device.

### General procedure

**Figure 3:**
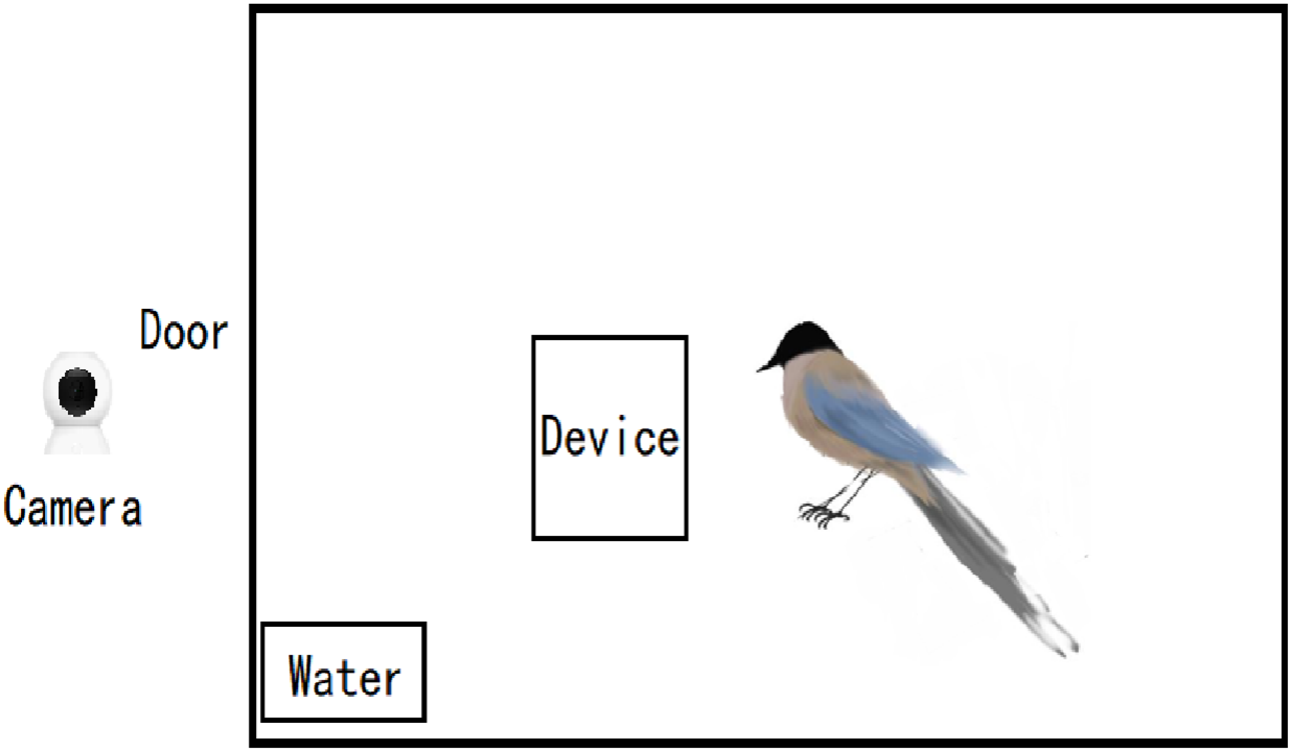
Schematic of experimental area.

The six individuals that completed the training were included in the six subsequent experiments. In each experiment, two mealworms were provided as rewards. In the subsequent experiments, regularly shaped rubber, foam, and plastic products were used instead of irregularly shaped natural stones (Fig. 2). Prior to the experiments, the maximum distance their beaks could reach the reward in the tube was measured to determine the initial water levels in the experiments. At least 10 habituations were conducted on each bird, that is, they were rewarded from different heights of the tube. As soon as the birds became accustomed to feeding in the tube (they got rewards from the top of the tube in 20 s), the experiments were commenced. Each experiment was repeated 20 times. The tubes used in experiment 1 and experiments 4–6 had a pseudorandom form, that is, the objects were placed not more than twice on the same side. By comparison, the objects used in experiments 2 and 3 were placed on both sides of the tube. In all experiments, the Azure-winged magpies’ choices (order and attributes) of tubes or objects were recorded using a camera (Xiaomi, MJSXJ03CM). In experiments 1–6, the entrance of the tube was modified to allow only the beak and not the head. “Choice” was defined as the Azure-winged magpie picked up with its beak and threw object into the tube. In some cases, the subject retrieved the object that had been put into the tube; hence, the number of selections was greater than the total number of objects. The Azure-winged magpies were placed in the experimental cage in advance. After each trial, the device was reset and put into the cage for the next trial. In experiments 1 and 4–6, the subjects were given 30 s to observe tubes, after which the objects were put in the cage. In experiments 2 and 3, the tube and the object were put at the same time. The experiments were ended after the magpies were rewarded or had left the experimental device for ≥ 30 s. The schematic of the experimental cage is illustrated in Fig. 3.

### Statistical analysis

The test employed almost exclusively in the previous studies is a two-tailed binomial test to detect departure from random choice (i.e. a probability of success different from 0.5)^1,4,5,7,13,30^. Given that the commonly used statistical methods cannot distinguish between the initial performance of the Azure-winged magpies and the trial-and-error learning that might have occurred during the experiments, and a single analysis of individuals may overestimate or underestimate the level of the group, we combined the choices of all individuals instead of analyzing them separately to evaluate whether the group can succeed in Aesop’s fable paradigm^18^. We fitted a generalized additive model (GAM) with selection (functional and nonfunctional) as dependent variables and trials as independent variables^31^. Wilcoxon test was used to test the first third and fifth trials, and the last third and fifth trials. The model could establish if trial-and-error learning occurred when the Azure-winged magpies completed the tasks and estimate their initial preference. Data were analyzed via a GAM model in R 4.0.2 (package: mgcv). *P* values ≤ 0.05 were considered statistically significant. For comparison, data on New Caledonian crows^1^ were also analyzed.

### Specific procedure and materials

#### Experiment 1: Water-filled tubes versus sand-filled tubes

**Figure 4:**
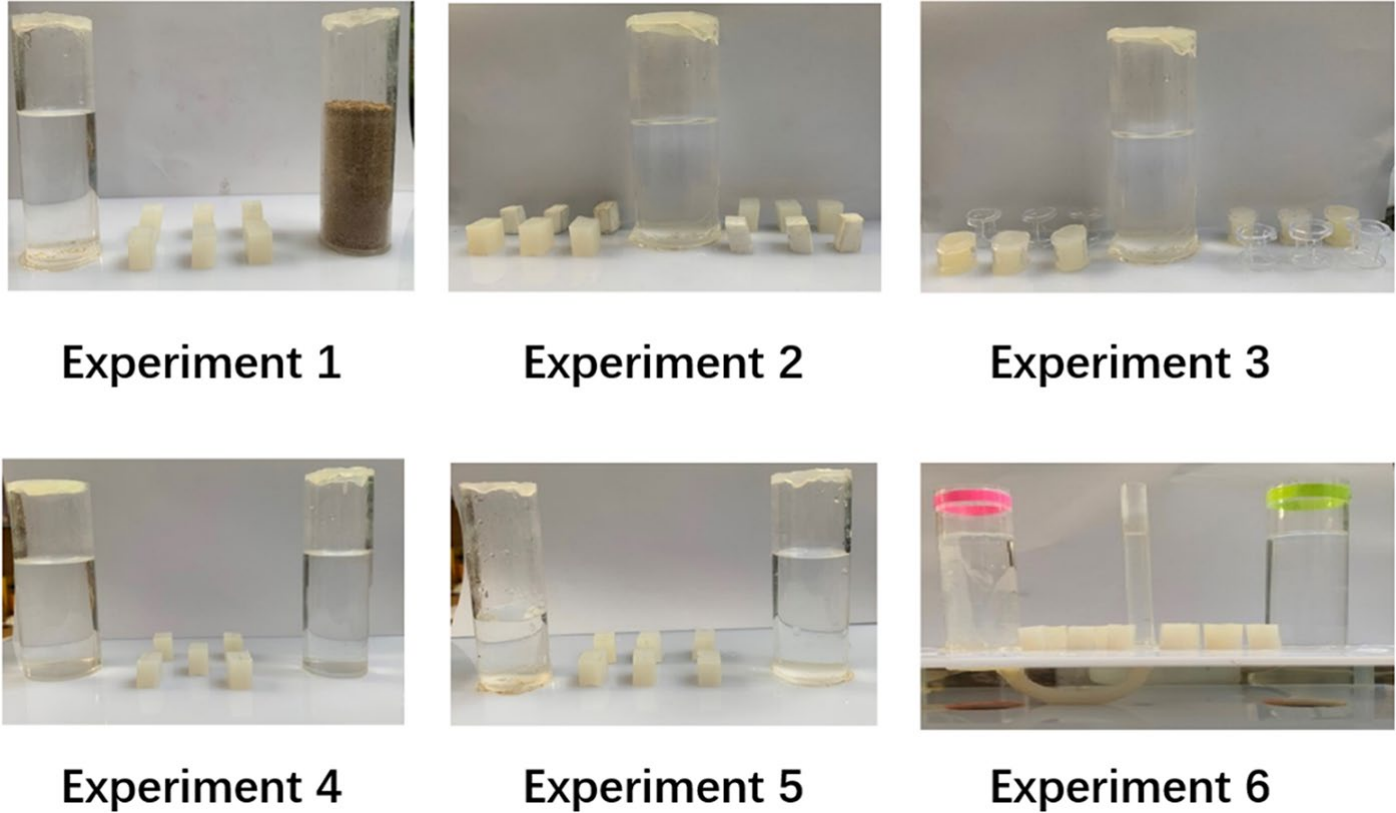
Schematic of the devices and objects used in experiments 1–6. The connection of U-shaped tubes in experiment 6 was obscured to the magpies. During the experiments, each experimental device was placed in the middle of the experimental cage.

 Two identical and transparent acrylic tubes (2 mm thickness × 35 mm outer diameter × 110 mm height) were fixed 110 mm apart on an acrylic plate (300 mm × 200 mm × 5 mm, the same below). The two tubes were separately filled with sand and water of equal level (each Azure-winged magpie had a corresponding sand and water level), and six rubber blocks were placed between the two tubes (Fig. 4). The rubber block raised the water level by 4 mm when it was put into the water-filled tube but did not change the sand level when put into the sand-filled tube. At least three rubber blocks were required to obtain the reward.

#### Experiment 2: Heavy objects versus light objects

 A separate transparent acrylic tube (2 mm thickness × 45 mm outer diameter × 110 mm) was fixed on another acrylic plate. The water level was 65–70 mm of the height of the tube, and 12 rubber blocks and foam blocks (six for each type) of the same size were placed on each side (3 rubber blocks and 3 foam blocks were presented together) of the tube (Fig. 4). The water level raised by 2 mm when the rubber block was inserted but about 0.1 mm when a foam block was inserted. At least four rubber blocks were required to obtain the reward.

#### Experiment 3: Solid objects versus hollow objects

 Another transparent acrylic tube (2 mm thickness × 45 mm outer diameter × 110 mm height) was fixed on another acrylic plate. The water level was 60–65 mm of the height of the tube, and 12 similar solid and hollow cylinders (six for each type) were placed on each side (3 solid cylinders and hollow cylinders were presented together) of the tube in sequence (Fig. 4). The water level raised by 3 mm when the solid cylinder was put into the tube, whereas the level raised by 0.5 mm only when the hollow cylinder was inserted. At least four solid cylinders were required to obtain the reward.

#### Experiment 4: Tubes of different diameters with the same water level

 Two transparent acrylic tubes with different outer diameters (35 and 45 mm outer diameter × 2 mm thickness × 110 mm height) were separately fixed 110 mm apart on an acrylic plate. The two tubes were filled with equal amounts of water (up to 60 mm in the tube), and five rubber blocks were placed in the middle of the two tubes (Fig. 4). The water level of small outer diameter tube raised by 4 mm when the rubber block was put into the tube, whereas the level of large outer diameter tube only increased by 1 mm when the rubber block was put into the tube. Notably, the reward could only be obtained if rubber blocks were inserted into the tubes with a small outer diameter. At least four rubber blocks were required to obtain the reward.

#### Experiment 5: Tubes with high water level versus tubes with low water level

 Two identical transparent acrylic tubes (2 mm thickness × 35 mm outer diameter × 110 mm height) were separately fixed 110 mm apart on an acrylic plate. The difference in water level between the two tubes was 30 mm. Six rubber blocks were placed in the middle of the two tubes (Fig. 4). The water level raised by 4 mm when the rubber blocks were put into the tubes. Notably, the reward could only be obtained if the rubber blocks were put in the tubes with a high water level. At least four rubber blocks were required to obtain the reward.

#### Experiment 6: U-shaped tubes

 Two identical transparent acrylic tubes (2 mm thickness × 35 mm outer diameter × 110 mm height) and a transparent plastic tube (10 mm outer diameter × 0.2 mm thickness × 110 mm height) were fixed side by side on an acrylic plate (300 mm × 200 mm × 5 mm). The plastic tube was placed in the middle of the two acrylic tubes with a distance of 50 mm between them. Five rubber blocks (10 in total) were placed between the plastic tube and the acrylic tubes. One acrylic tube was connected to a U-shaped plastic tube at the bottom (not visible to the magpies). To attract more attention to the functional tubes, we marked the orifice of the acrylic tube connected to the plastic tube with red and the orifice of the other acrylic tube with green. The reward was put inside the plastic tube. The water level in all tubes was equal. On the rubber blocks could be put into the acrylic tubes because the diameter of the plastic tube was smaller than any of the edges of the rubber blocks. The water level could only be raised by placing the blocks into the acrylic tube connected to the plastic tube (Fig. 4). The water level raised by 2.1 mm when the rubber blocks were put into the tubes. At least four rubber blocks were required to obtain the reward.

## Results

**Figure 5:**
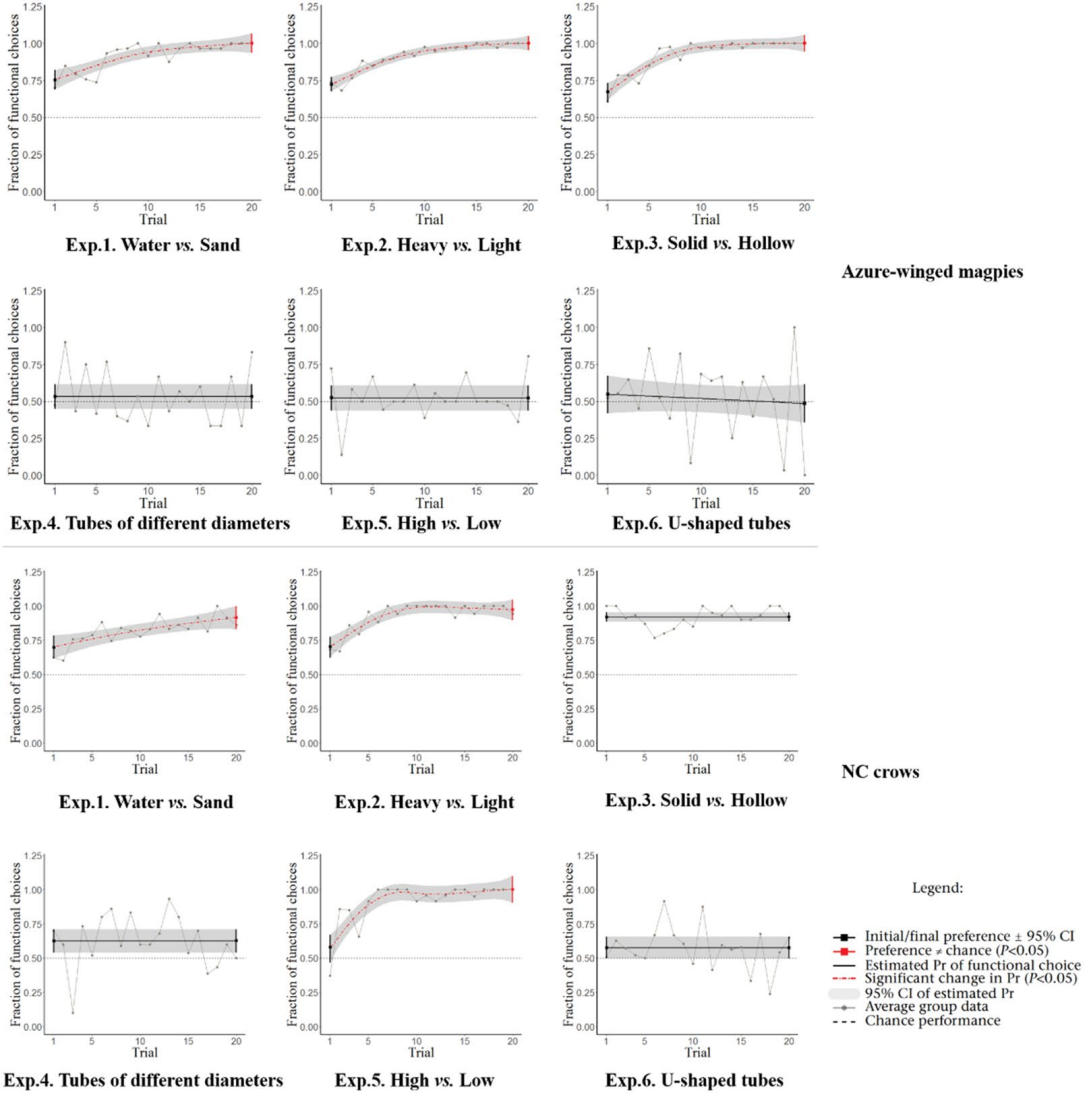
Experimental results of Azure-winged magpies and New Caledonian (NC) crows. Dotted lines represent the probability that the functional option will be selected. Gray dots denote the proportion of functional options selected by all subjects in each trial. Solid lines indicate the probability of a functional choice as estimated by GAM regression (see Methods). Lines are depicted in red and dots are shown as dashes when the probability significantly increases; otherwise, the lines are shown as black only. The first and last probability estimates are represented by solid dots. The dots are marked as red if there is a significant difference between the first and last probability estimates and the dotted line value (*P* < 0.05); otherwise, the dots are shown as black. The gray area represents the 95% confidence interval of the probability estimate represented by the implementation.

Seven Azure-winged magpies were included in the experiments. As previously mentioned, K failed to complete the transition from phase I to phase II in the initial training. Thus, only six individuals actually participated in the subsequent experiments. All six individuals completed experiments 1–5, but only three individuals completed experiment 6. The three other individuals lost interest in experiment 6 because their motivation waned. The results of statistical analysis are shown in Fig. 5 and summarized in Table 1. Examples of each experiment and experimental data for each individual in each experiment are provided in the supplementary materials.

**Table 1:** Proportion of functional objects selected by all subjects in four stages.

	Exp.1		Exp.2		Exp.3		Exp.4		Exp.5		Exp.6	
	A	B	A	B	A	B	A	B	A	B	A	B
First 3 trials	0.779	0.57	0.729	0.545	0.726	0.929*	0.602	0.269	0.481	0.582	0.575	0.593
First 5 trials	0.767	0.629	0.745	0.657	0.790	0.899*	0.605	0.327	0.438	0.649	0.601	0.585
Last 3 trials	1.000*	0.902*	1.000*	0.976*	1.000*	0.969*	0.611	0.290	0.546	1.000*	0.344	0.377
Last 5 trials	0.987*	0.856*	0.994*	0.972*	1.000*	0.929*	0.500	0.309	0.528	0.986*	0.443	0.408

Proportion of functional objects selected by all subjects in the first third and fifth trials, and the last third and fifth trials.

A) Azure-winged magpies, B) New Caledonian crows.

Significant results are marked with * (P < 0.05).

**Experiment 1: Water-filled tubes versus sand-filled tubes.** Water-filled tubes were designated as the functional option in this experiment. In the first trial, the magpies did not show preference for water-filled tubes (W = 4.5, P_trial1_ = 0.61). However, as the number of trials increased, they gradually showed preference for these tubes (W = 6, P_trial20_ = 0.04). They passed the experiment by trial-and-error learning.

**Experiment 2: Heavy objects versus light objects.** Heavy objects were assigned as the functional option in this experiment. In the first trial, the magpies did not show preference for heavy objects (W = 6, P_trial1_ = 0.29). However, as the number of trials increased, they gradually showed preference for these objects (W = 6, P_trial20_ = 0.04). They passed the experiment by trial-and-error learning.

**Experiment 3: Solid objects versus hollow objects.** Solid objects were selected as the functional option in this experiment. In the first trial, the magpies did not show preference for solid objects (W = 6, P_trial1_ = 0.19). However, as the number of trials increased, they gradually showed preference for these objects (W = 6, P_trial20_ = 0.04). They passed the experiment by trial-and-error learning.

**Experiment 4: Tubes of different diameters with the same water level.** Narrow tubes were the functional option in this experiment. The magpies did not show preference for narrow tubes in the first and last trials (W = 3, P_trial1_ = 1.00; W = 5, P_trial20_ = 0.35). They failed to pass the experiment.

**Experiment 5: Tubes with high water level versus tubes with low water level.** High water level was the functional option in this experiment. The magpies did not show preference for high water level in the first and last trials (W = 5, P_trial1_ = 0.43; W = 5, P_trial20_ = 0.41). They failed to pass the experiment.

**Experiment 6: U-shaped tubes.** The tube marked with red stripes (hereinafter referred to as red tube) was designated as the functional option in this experiment. The magpies did not show preference for the red tube in the first and last trials (W = 1.5, P_trial1_ = 1.00, W = 0; P_trial20_ = 0.25). They failed to pass the experiment.

## Discussion

The present results on Azure-winged magpies in Aesop’s fable paradigm were comparable with those on New Caledonian crows^1,5^. Nevertheless, several differences were noted (a further comparison is provided below). The Azure-winged magpies preferred water-filled tubes over sand-filled tubes, heavy objects over light objects, and solid objects over hollow objects. However, they failed to distinguish between narrow and wide tubes, high and low water level tubes, and were unsuccessful in the experiment with U-shaped tubes. Our study shows that Azure-winged magpies passed experiments 1–3 by trial-and-error learning, but failed to pass experiments 4–5.

In the wild, the primary food items of Azure-winged magpies are insects, such as bugs (*Hemiptera*) and beetles and their larvae (*Coleoptera*), as well as plant fruits and seeds. Thus, it seems that they do not naturally feed by using stones. Therefore, we trained seven Azure-winged magpies under laboratory conditions. We call this training “operant conditioning”. The purpose of this training was to make the Azure-winged magpies to understand that behavior of throwing objects into the tube is the only way to obtain rewards. In other words, other objects aside from stones can be used. Therefore, no stone was used in experiments 1–6 that it can reduce object–bias interference. From the perspective of cognitive psychology, we hoped that the Azure-winged magpies would exhibit the ability of “transfer of training”, that is, individuals can solve similar problems (getting rewards from the tube) on the basis of their previous experience-behavior of throwing objects into the tube is the way to obtain rewards. Transfer of training can be divided into positive transfer and negative transfer. Positive transfer states that previous experience (getting rewards from the tube by throwing objects into the tube, same below) can help individuals quickly solve current problems, whereas negative transfer denotes that previous experience will interfere or reduce the individual’s ability to solve problems^32^. In the present study, both types were observed in the experiments.

In experiments 1–3, their choices of functional options were significant in the last third and fifth trials but not in the first third and fifth trials (Table 1). Furthermore, the Azure-winged magpies failed to learn to differentiate between the two tubes based on the different water levels, that is, they did not show preference for tubes according to water level. By contrast, the New Caledonian crows showed preference for tubes with a high water level in experiment 5 (Table 1). Although the Azure-winged magpies did not show preference in experiment 5, this result did not necessarily mean that the Azure-winged magpies are inferior to the New Caledonian crows in solving problems because our experiment 5 was more complicated than the previous study. That is, the appearance and size of the two tubes were the same in experiment 5. In the experiment 5 of Jelbert, et al.^1^, the appearance and size of the tubes were different. Notably, the New Caledonian crows showed preference in the first third, and first fifth trials in experiment 3 (Fig. 5 and Table 1). This result can be explained by the object preference hypothesis. However, in our improved Aesop’s fable paradigm, the Azure-winged magpies did not show initial preference in the first third, and first fifth trials (Fig. 5 and Table 1).

Although Aesop’s fable paradigm did not prove that the magpies understood causality well, experiments 1–5 had causal cues, whereas experiment 6 had no causal cues (the connection of U-shaped tubes was obscured). Therefore, we consider the former as causal tasks and the latter as arbitrary tasks. Jelbert, et al.^13^ argued that species of the family Corvidae can pass some causal cues but not arbitrary cues because they notice and understand causal cues. In other words, subjects can learn causal cues faster than arbitrary cues. Bhat and Mohan’s work emphasized that reasoning and learning always have to go hand-in-hand and grow cumulatively and continuously in lifetime of a learner, be it a natural or an artificial cognitive agent^21^. Therefore, on the basis of Aesop’s fable paradigm, we speculate that the Azure-winged magpies used causal clues.

**Figure 6:**
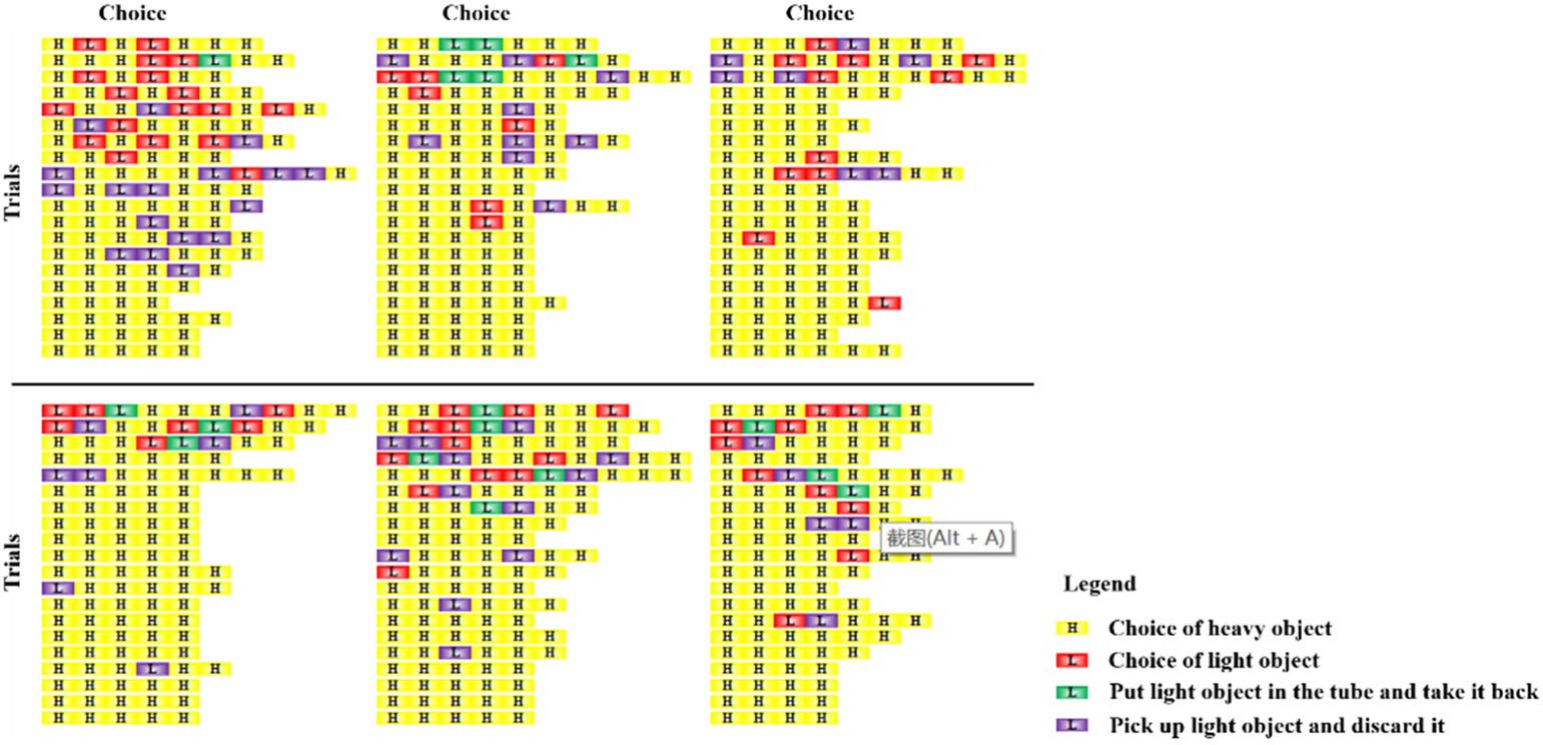
Azure-winged magpies’ choices of objects in experiment 2.

In this study, we improved the experimental methods. In the initial training phase, we used natural stones, whereas the objects utilized in experiments 1–6 were different from natural stones in terms of size, weight, and appearance (Fig. 2). Notably, transfer of training of initial training from experiment 1 is positive transfer. In experiments 2 and 3, the Azure-winged magpies were forced to choose the functional objects from all the objects we provided. Therefore, if the magpies only relied on behavior of throwing objects into the tube is the way to obtain rewards, then they would not have shown any preference for the functional objects, that is, whether the Azure-winged magpies throws functional or non-functional objects into the tube were rewarded. Actually, at the beginning of the experiment, Azure-winged magpies just threw a certain number of objects (whether functional or non-functional) into the tube and tried to get the rewards (transfer of training). However, as the number of trials increased, they gradually showed preference the functional objects, and the probability of this preference substantially increased. Notably, the correct rate of the last third and fifth trials was over 90%. In the 20th trial, the probability even reached 100%. Thus, the object–bias hypothesis alone seems inadequate to explain the success of the magpies in experiments 2 and 3. Furthermore, the rate of discarding light objects after the fifth trial was close to 100% in experiment 2 (Fig. 6). Therefore, the magpies at least relied on trial-and-error learning in the experiment, indicating the occurrence of negative transfer among experiment 1 and experiment 2.

Jelbert, et al.^13^ claimed that corvids can notice changes in water level. In the present study, the magpies passed the water-filled tube *vs.* sand-filled tubes experiment, consistent with the explanation of the feedback hypothesis. The magpies performed well in experiments 1–3 in causal tasks but did not prefer functional options in experiments 4 and 5. Except for the sand-filled tube task in experiment 1, the causal tasks in this study changed the position of the reward in the tube after the object was put into the tube for other tasks (the nonfunctional option was not completely nonfunctional). According to the feedback hypothesis, no strong preference for the functional options should be observed in experiments 2 and 3 because all objects provide feedback for subjects. However, the Azure-winged magpies showed notable preference for the functional options in experiments 2 and 3 in the last third and fifth trials. Therefore, the feedback hypothesis is insufficient to explain the success of experiments 2 and 3. In experiments 4 and 5, although the narrow tube was more functional than the wide tube, and the high water level was more functional than the low water level, the Azure-winged magpies did not show any preference. Birds have excellent eyesight^33^. Hence, Azure-winged magpies should be able to distinguish the difference between wide and narrow tubes and between high and low water levels. However, they don't show the behavior of taking advantage of these differences, that is, preference for functional options. We speculate that the spatial cognitive ability of Azure-winged magpies is not perfect. In experiments 4 and 5, the magpies kept filling the same tube with rubber blocks until all blocks were used. We speculate that the Azure-winged magpies noticed that the position of the reward changed when they put the object into the tube. Nevertheless, in these experiments, the Azure-winged magpies continually repeated the steps simply relied on feedback to obtain rewards without utilizing the difference between the tubes.

There may be another reason why Azure-winged magpie had no preference for functional options in the last three tasks. The analogical problem solving—the process by which current problems are addressed by using the lessons they learned in resolving previous problems—might have affected their performance^34^. The following factors might have also influenced the performance of the Azure-winged magpies in the experiments. First, superficial similarity—details not related to the solution are the same in the two problems (rewards are floating on water); second, structural similarity—the causality of some major components in two problems is the same (throwing an object into the tube will cause the reward to shift); third, procedural similarity—the procedures for translating problem-solving principles into specific operations are the same in two problems (rewards can only be obtained when objects are dropped into the water). Experiments 1–6 were performed in strict order, and the subsequent tasks were conducted only after the previous task was completed. Hence, the magpies likely drew on their experience in solving problems in experiments 1–3 to solve the problems presented to them in subsequent experiments. In addition to experiment 1, the Azure-winged magpies were rewarded by putting a certain number of functional objects into the tube in experiments 2 and 3. Therefore, in the later task (selecting functional tubes), the Azure-winged magpies may be influenced by previous experiments, that is, continuously putting objects into the tube can obtain rewards.

We also analyzed the data of Jelbert, et al.^1^ on New Caledonian crows (Table 1 and Fig. 5). The performance of the crows in the task on water-filled tubes *vs.* sand-filled tubes was not good as their performance in other tasks because the crows committed more mistakes in this task^1^. The opposite result was observed for Azure-winged magpies. The magpies quickly adjusted their behavior and performed very well in the first three tasks. Therefore, to some extent, the flexibility in the behavior of Azure-winged magpies is stronger than that of New Caledonian crows. Although Azure-winged magpies failed in experiment 4, their performance in this task—they put a limited number of stones into the same tube—demonstrated that they relied on feedback. As mentioned previously, the Azure-winged magpies could obtain the reward as long as they were given enough objects. The Azure-winged magpies behaved similarly to other corvids in the tasks that they passed^1,7,8^. The Azure-winged magpies stopped throwing objects into the tube after obtaining the reward, but they continued putting more objects into the same tube without trying to obtain the reward in the tasks they failed. The Azure-winged magpies usually needed three to five objects to obtain the reward. In the last tenth trial throughout the experiments, they did not try to obtain the reward in advance until they had put three or more objects. These observations showed that Azure-winged magpies noticed the displacement of the water. Nevertheless, in these experiments, the Azure-winged magpies continually repeated the steps simply relied on feedback to obtain rewards without utilizing the difference of displacement of the water.

Like other corvids, the Azure-winged magpies failed to pass arbitrary tasks^1,5,7^. Arbitrary tasks are more difficult than causal tasks because they require more advanced cognitive abilities, such as reasoning, and because their causal clues are hidden and counterintuitive. Intuitive theories for unobservable causal mechanisms undoubtedly play an important role in human causal cognition^2^. This task can only be accomplished by children over 7 years old^12^. Azure-winged magpies can theoretically pass this task in two ways: (1) via feedback and (2) by inferring the hidden connection of U-shaped tubes. Feedback should allow the Azure-winged magpies to associate the red tubes with rewards after trial and error. However, the magpies did not show preference for red tubes in the experiments. The multiple-resource theory may explain why the magpies failed to use feedback in experiment 6. This hypothesis states that similar tasks compete for the same specific resources, thereby causing interference^35^. The experimental device used herein was too large relative to the subjects, and the subjects did not notice that the position of the reward in the plastic tube changed after the object was put into the acrylic tube^1^. Both the object-catching behavior and the change in the position of the reward required visual participation (competing for visual resources). Consequently, the Azure-winged magpies did not notice in which tube the position of the reward changed after the object was thrown. All Azure-winged magpies in this task immediately attempted to obtain the reward from the plastic tube during the first trial, and some individuals even tried to put the object into the plastic tube all the time. Why did they do this? The tube where the rewards could be obtained in experiments 1–5 was the same tube where the stone was thrown. Therefore, this task was counterintuitive compared with the first five tasks. The Azure-winged magpies relied on their experience that they could be rewarded when they put objects into the tube and applied it to the current task. Thus, if the first five experiments could be regarded as training, then the magpies had a negative transfer in experiment 6. The New Caledonian crow named Kitty solved this task. However, it failed in the task on U-shaped tube with unobscured connection possibly because it only noticed which colored tube could cause a change in the position of the reward rather than inferred the hidden connection^5^. Consequently, the unique non-human subjects who passed were only able to relate the color to the correct tube, not to infer the hidden connection of U-shaped tubes.

In general, our experimental results demonstrated that Azure-winged magpies have a certain cognitive ability. They noticed water displacement, could distinguish the difference between sinking and floating objects, and determine the difference between solid and hollow objects. Although we cannot completely rule out the object-bias hypothesis, we believe that object-bias has little effect on Azure-winged magpies in this study. We can conclude that the magpies completed part of the tasks by trial-and error- learning. These results confirmed that Azure-winged magpies have a cognitive ability similar to that of other corvids. However, the experiments failed to establish that Azure-winged magpies have an understanding of causality. Nevertheless, from the perspective of cognitive psychology, the results showed that the magpies have the ability of training transfer and analogical problem solving. The performance of the Azure-winged magpies in string-pulling tasks^24,27^ and mirror tasks^28^ also showed that they have a certain, although limited, spatial cognitive ability. We should emphasize that interference among experiments should be avoided in designing experiments to test cognitive ability. In a follow-up study, we will design more rigorous experiments that will explore in greater detail the various cognitive abilities of Azure-winged magpies, such as space cognition and social cognition, and to construct a structural cognitive structure map of this bird species.

## Supplementary Information

Supplementary Legends.

Supplementary Data.

Supplementary Video.
