# Supplementary material for: Performance of Azure-winged magpies in Aesop’s fable paradigm
Source: Sci Rep. 2021 Jan 12;11:804. doi: 10.1038/s41598-020-80452-5 (PMC7804021; doi:10.1038/s41598-020-80452-5)
Supplement: Supplementary file 1 — Supplementary Legends. [file 41598_2020_80452_MOESM1_ESM.docx]

**Performance of** **Azure-winged magpies in Aesop’s fable paradigm**

Yigui Zhang, Cong Yu, Lixin Chen, Zhongqiu Li*

(Lab of Animal Behavior & Conservation, School of Life Sciences, Nanjing University, Nanjing, Jiangsu, 210023, China)

Corresponding author: lizq@nju.edu.cn

**legends of supplementary material：**

**Experiment 1：**

W：Water

S: Sand

**Experiment 2：**

H: Heavy

L: Light

**Experiment 3：**

S: Solid

H: Hollow

**Experiment 4：**

N: Narrow

W: Wide

**Experiment 5：**

H: High

L: Low

**Experiment 6：**

R: Red

G: Green
